# Oral nystatin prophylaxis in surgical/trauma ICU patients: a randomised clinical trial

**DOI:** 10.1186/cc11300

**Published:** 2012-04-10

**Authors:** Mariateresa Giglio, Giuseppina Caggiano, Lidia Dalfino, Nicola Brienza, Ilaria Alicino, Aurelia Sgobio, Antonella Favale, Caterina Coretti, Maria Teresa Montagna, Francesco Bruno, Filomena Puntillo

**Affiliations:** 1 Anaesthesia and Intensive Care Unit, Department of Emergency and Organ Transplantation, University of Bari, Piazza G. Cesare 11, Bari 70124, Italy; 2 Department of Biomedical Science and Human Oncology, Hygiene Section, University of Bari, Piazza G. Cesare 11, Bari 70124, Italy

## Abstract

**Introduction:**

*Candida *prophylaxis in ICU is still a matter of debate. Oral chemoprophylaxis has been advocated to reduce the incidence of *Candida *colonisation and infection.

**Methods:**

We performed a randomised trial studying a single drug (nystatin) versus control in surgical/trauma ICU patients. Multiple-site testing for fungi was performed in each patient on ICU admission (T0) and subsequently every 3 days (T3, T6, T9, and so forth). The primary evaluation criterion was the time course of the corrected colonisation index.

**Results:**

Ninety-nine patients were enrolled. At admission, 69 patients exhibited *Candida *colonisation: the most frequently colonised body sites were the stomach and the pharynx. The most frequent isolated species was *Candida albicans*. The corrected colonisation index was similar in the two groups at T0 (*P *= 0.36), while a significant statistical difference was observed between the treatment and control groups at T6 (median 0.14 and 0.33, respectively; *P *= 0.0016), at T9 (median 0.00 and 0.28, respectively; *P *= 0.0001), at T12 (median 0.00 and 0.41, respectively; *P *= 0.0008), and at T15 (median 0.00 and 0.42, respectively; *P <*0.0003). The same results were obtained in the subgroup of patients already colonised at ICU admission.

**Conclusion:**

This trial shows that nystatin prophylaxis significantly reduces fungal colonisation in surgical/trauma ICU patients, even if already colonised.

**Trial registration:**

ClinicalTrials.gov: NCT01495039

## Introduction

The challenge posed by nosocomial fungal infections in critically ill patients has become increasingly apparent over the past 20 years. *Candida *species are now among the leading pathogens in ICUs in both Europe and the United States [1-6]. The incidence of nosocomial candidaemia has dramatically increased and has been associated with high overall (35 to 80%) and attributable (30 to 40%) mortality [3]. Moreover, it has been reported that the length of stay of critically ill patients who survived candidaemia was prolonged from 8 to 30 days with a significant increase of nursing workload [7]. Candidaemic patients needed a prolongation of mechanical ventilatory support of 10 days [8]. Candidaemia is associated with high morbidity, high mortality, and the significant use of additional resources.

Colonisation by *Candida *species is the leading risk factor for infection, and several elements support the assumption that multiple- site colonisation is a prerequisite for subsequent infection [9-12]. Several risk factors can promote further invasion with possible secondary haematogenous dissemination; among these risk factors, surgical procedures seem to play a key role [13].

Assuming the risk of death is similar in multiple-site colonised surgical patients and in those with proven candidiasis [14], several studies have focused on the degree of colonisation and screening. Pittet and colleagues established the degree of colonisation with the *Candida *colonisation index (CI) and found a strong correlation between colonisation intensity (that is, CI > 0.5) and invasive infections [9]. The same authors showed that considering heavy colonised body sites with the corrected colonisation index (CCI) enhances the discriminatory power of the CI, with higher (100%) sensitivity, specificity, positive predictive, and negative predictive values than the CI [9].

For these reasons, oral chemoprophylaxis has been advocated for ICU patients, and in particular for surgical ICU patients, with the aim to reduce the incidence of heavy colonisation and infection [15], although whether this approach should be implemented remains controversial. Oral nystatin prophylaxis efficiently prevented *Candida *species colonisation both in medical and surgical patients that were not colonised at admission to the ICU [16]. Since colonisation can be observed on admission in up to 50% of ICU patients [17,18] a considerable cohort was excluded, making these positive results not applicable to all ICU populations.

We therefore decided to perform a randomised controlled trial to evaluate the time course of CCI in a surgical/trauma ICU population, including those colonised at admission, undergoing nystatin prophylaxis.

## Materials and methods

We performed a randomised, open-label, single-centre study with blinded assessment of the objective primary evaluation criterion. We studied a single drug (nystatin) versus control. The study was approved by the Ethics Committee of the Policlinico Hospital, Bari, Italy and was conducted in accordance with the Helsinki Declaration (ClinicalTrials.gov: NCT01495039).

The study was performed from November 2008 to August 2009 (date of final data collection for the primary outcome measure). The primary evaluation criterion was the time course of the CCI; the secondary evaluation criterion was occurrence of a fungal infection.

### Study population

The patients or their next of kin provided informed consent for participation in the study. The inclusion criteria were: surgical patients admitted to our ICU > 18 years of age and expected to require invasive mechanical ventilation for more than 48 hours. The exclusion criteria were: pregnancy, proven *Candida *infection, prophylactic or curative antifungal treatment within the last 2 months, contraindication to oral drug administration, known allergy to nystatin or its derivatives, and prior inclusion in the study.

Reasons for admission, demographic characteristics, immune status, and the Sequential Organ Failure Assessment score were recorded on admission. The duration of mechanical ventilation, the duration of antibiotic and corticosteroid therapy, the length of stay in the ICU, the route of nutrition (that is, enteral vs. parenteral), and mortality were also recorded. In cases of residual gastric volume > 500 ml/24 hours or vomiting, the patient was excluded from the analysis. Risk factors for *Candida *infection were identified and recorded as previously suggested (that is, diabetes, previous antibiotic and corticosteroid therapy or dialysis, central venous catheter, parenteral nutrition, multiple transfusions, pancreatitis, chronic renal failure, immunosuppressive therapy other than steroids, leucopenia (white blood cells < 4,000/mm)) [19]. Patients were randomised to one of the two study groups, according to a randomisation sealed envelope opened on admission to the ICU, to receive either systematic nystatin prophylaxis (2 × 10^6 ^U/day administered three times daily via the nasogastric tube; group N) or no nystatin prophylaxis as control (group C).

### Definitions and mycological assessment

Multiple-site testing for fungi included tracheal secretions, swab, stomach contents, pharyngeal, rectal and groin skinfold swabs, urine, and blood. These tests were performed in each patient at ICU admission (T0) and subsequently every 3 days throughout the ICU stay (T3, T6, T9, and so forth). The specimens were placed in a dry medium and taken to the Mycology Laboratory. Group assignment was not indicated on the specimens, so the mycologists were therefore blinded to treatment allocation. Each specimen was directly microscopically examined and cultured on Sabouraud media. The attending physicians were not aware of the results of the colonisation samples, and therefore no empirical or pre-emptive antifungal therapy was in place in enrolled patients.

Colonisation was assessed for each body site specimen, and yeasts were identified. Fungal colonisation was defined as the presence of the same yeast on one or more of the six distinct body sites tested (blood sample excepted). The CI was calculated for each multiple-site testing as the ratio between the number of distinct body sites colonised by *Candida *species (except blood) and the total number of sites tested. The CCI was calculated for each time point as the product of the CI multiplied by the ratio of the number of distinct nonblood body sites showing heavy growth to the total of body sites growing *Candida *species. Fungal infection was defined as either the presence of candidaemia or the identification of *Candida *species in a normally sterile body site, associated with severe sepsis and negative tests for bacteria. During the study period, adverse events related to the study drug were monitored (that is, diarrhoea, nausea, vomiting, intestinal pain, urticarial skin reactions).

### Outcome measures and statistical analysis

Continuous variables are expressed as the mean ± standard deviation or median (interquartile range). Categorical variables were compared by chi-square test or Fisher's exact test. Student's *t *test was used to compare normally distributed continuous variables, and the Mann-Whitney U test was used to analyse variables not normally distributed. All *P *values were two-tailed. Statistical significance was set at *P *≤ 0.05. For an estimated rate of fungal colonisation reaching approximately 60% in ICU patients, 49 patients per group had to be enrolled in the study to show a 50% reduction in fungal colonisation, with an α error of 5% and a β error of 20%.

## Results

**Figure 1:**
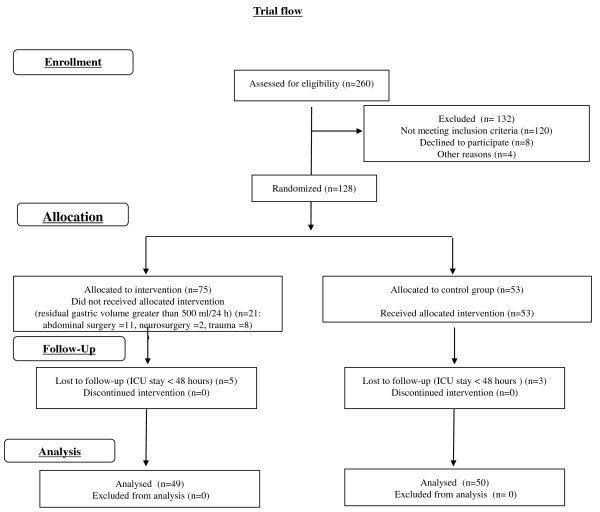
**Flow diagram of progress through the phases of this randomised trial for the two groups**.

Of 260 patients assessed for eligibility, 128 were randomised to the two study groups. Of these randomised patients, 99 completed the study (61 men, 38 women): 49 patients were randomised to group N and 50 patients to group C (see Figure 1 for trial flow).

The two groups were well matched in terms of age, sex, baseline morbidity, risk factors for *Candida *infection, and reason for admission to the ICU. The mean age was 56 ± 20 years and the mean Sequential Organ Failure Assessment score was 7 ± 2. The reason for ICU admission was abdominal surgery in 15 patients, neurosurgery in 45 patients, and trauma in 39 patients. The most frequent risk factors for *Candida *infection were central venous catheters (*n *= 99), followed by antibiotic therapy (*n *= 82) and parenteral nutrition (*n *= 56). The duration of mechanical ventilation as well as the ICU stay were similar between the two groups (Table 1).

**Table 1:** Demographic characteristics of patients included in the two study groups

	Nystatin group (*n *= 49)	Control group (*n *= 50)	*P *value
Age (years)	54 ± 22	58 ± 19	0.27
Female/male	18/31	20/30	0.80
Sequential Organ Failure Assessment score	7 ± 2	7 ± 2	0.69
APACHE II score	19.5 ± 7	19.3 ± 8	0.89
Type of admission			
Abdominal surgery	6 (12%)	9 (18%)	0.42
Neurosurgery	19 (39%)	26 (52%)	0.19
Trauma	24 (49%)	15 (30%)	0.05
Duration of hospital stay before ICU admission (days)	2 ± 4	2 ± 4	0.6
Duration of mechanical ventilation (days)	13 ± 11	13 ± 12	0.93
Duration of ICU stay (days)	15 ± 14	15 ± 14	0.86
Risk factors			
Diabetes mellitus	13 (26%)	9 (18%)	0.3
Neutropenia (WBC < 4,000/mm^3^)	0	0	
Steroids (days -7 to 3)	11 (22%)	7 (14%)	0.96
Multiple transfusions	12 (24%)	13 (26%)	0.86
Pancreatitis (days -7 to 0)	0	2 (4%)	0.55
Chronic renal failure	0	1 (2%)	0.31
Immunosuppressive agents other than steroids (days -7 to 0)	0	0	
Dialysis (days 1 to 3)	0	1 (2%)	0.31
Total parenteral nutrition (days 1 to 3)	29 (59%)	27 (54%)	0.36
Central venous catheter (days 1 to 3)	49 (100%)	50 (100%)	
Antibiotic therapy (days 1 to 3)	40 (81%)	42 (84%)	0.63
Acute renal failure	0	0	
Mortality	11 (22%)	13 (26%)	0.68

Data presented as mean ± standard deviation or *n *(%). Risk factors are recorded as suggested by Ostrosky-Zeichner and colleagues [19]. APACHE, Acute Physiology and Chronic Health Evaluation; WBC, white blood cells.

**Figure 2:**
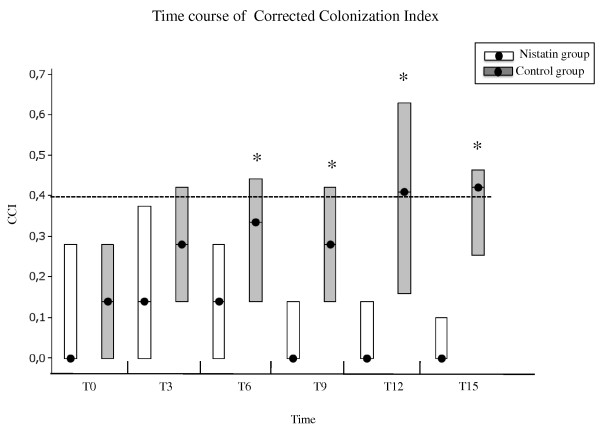
**Course of the corrected colonisation index over time**. Course of the corrected colonisation index (CCI) over time in the treatment group (white bars) and the control group (black bars). Illustrated are the daily median values (filled circles), and the 25th and 75th percentiles - that is, the interquartile range (borders of the box). Data in parentheses are the number of patients in each of the two study groups over time. *P <0.05.

At admission, 69 patients exhibited *Candida *colonisation (34 in group N and 35 in group C) while 30 patients were not colonised (15 patients in both groups). The most frequently colonised body sites were the stomach and the pharynx (58% and 47% of the total sample obtained, respectively), followed by the trachea (27%) and the rectum (20%). There were no differences between the two groups at this time. The most frequent isolated *Candida *species at T0 was *Candida albicans *(71%), followed by *Candida glabrata *(14%) and *Candida krusei *(1%). Figure 2 depicts the CCI for the two groups over time. The CCI was comparable in the two groups at T0 (*P *= 0.36), while a significant statistical difference was observed between group N and group C at T6 (median 0.14 and 0.33, respectively; *P *= 0.0016), at T9 (median 0.00 and 0.28, respectively; *P *= 0.0001), at T12 (median 0.00 and 0.41, respectively; *P *= 0.0008), and at T15 (median 0.00 and 0.42, respectively; *P <*0.0003).

**Figure 3:**
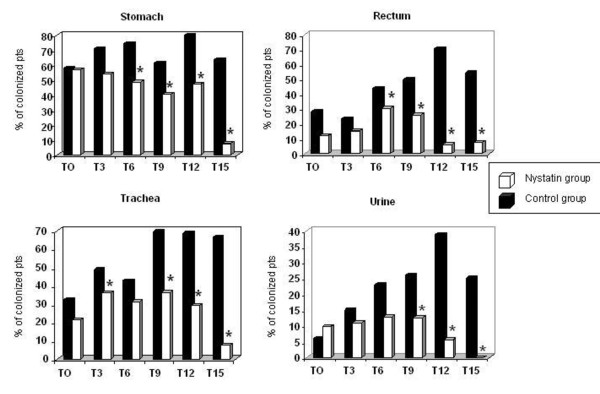
**Colonisation of different body sites**. Percentage of patients colonised during the study period in four different body sites: stomach, rectum, trachea, and urine. Black bars, control group; white bars, nystatin group. *P <0.05 between groups at every time (T0, T3, and so forth). Pts, patients.

During hospitalisation in the ICU the proportion of positive stomach samples significantly decreased in the treatment group (from 59 to 49%) as opposed to the control group (from 58 to 74%; *P <*0.00009) at T6, and this difference persisted over time. At the end of the study period, the percentage of positive rectum samples significantly decreased in group N (from 12 to 8%) while it increased from 28 to 55% in group C (*P <*0.0001). A significant reduction of positive urinary samples was also noticed in group N (from 10 to 0%) compared with group C (from 6 to 25%; *P <*0.016), No difference was detected in pharyngeal samples (Table 2 and Figure 3).

**Table 2:** Total and positive samples obtained in the two groups at each time-point for every site

Sample		T0	T3	T6	T9	T12	T15
Trachea	Positive samples in group C	16	18	15	16	11	6
	Positive samples in group N	11	16	12	11	5	1
	Total samples obtained	99	81	72	53	33	22
Stomach	Positive samples in group C	29	27	26	16	15	7
	Positive samples in group N	29	21	19	13	9	1
	Total samples obtained	99	86	72	57	37	24
Pharynx	Positive samples in group C	23	23	20	18	13	7
	Positive samples in group N	24	22	15	18	11	8
	Total samples obtained	95	73	65	53	33	23
Skin	Positive samples in group C	2	3	8	6	6	4
	Positive samples in group N	0	1	4	0	2	2
	Total samples obtained	100	86	72	58	37	28
Rectum	Positive samples in group C	13	8	15	13	12	6
	Positive samples in group N	6	7	12	8	1	1
	Total samples obtained	95	79	72	57	33	24
Urine	Positive samples in group C	3	6	8	7	7	3
	Positive samples in group N	5	5	5	4	1	0
	Total samples obtained	99	86	72	59	36	28
Blood	Positive samples in group C	0	0	0	0	0	0
	Positive samples in group N	0	0	0	0	0	0
	Total samples obtained	99	86	72	59	37	28

Group N, nystatin group; group C, control group.

**Figure 4:**
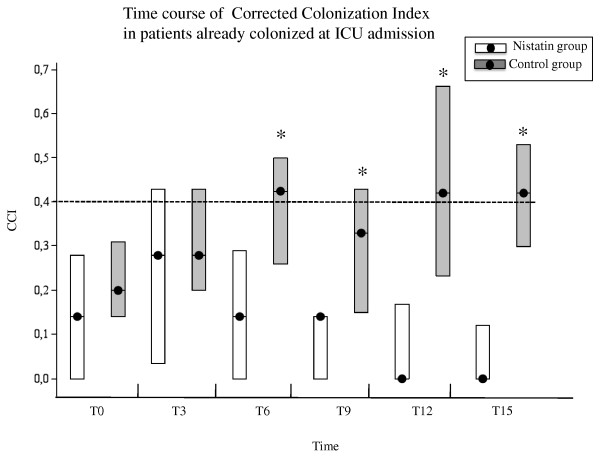
**Corrected colonisation index over time in patients already colonised at admission to the ICU**. Course of the corrected colonisation index (CCI) over time in the treatment group (white bars) and the control group (black bars), considering only patients who were already colonised at admission to the ICU. Illustrated are the daily median values (filled circles), and the 25th and 75th percentiles - that is, the interquartile range (borders of the box). Data in parentheses are the number of patients in each of the two study groups over time. *P <0.05.

Among patients colonised at admission, no statistical difference in CCI was found between the two groups at T0 and T3 (*P *= 0.26 and *P *= 0.18, respectively). At T6, however, group N showed a statistical significant reduction of CCI (median 0.14 in group N vs. 0.42 in group C, *P *= 0.0007), and this difference persisted at T9 (median 0.14 vs. 0.33, respectively; *P *= 0.0004), at T12 (median 0.00 vs. 0.42, respectively; *P *= 0.0005), and at T15 (median 0.00 vs. 0.42, respectively; *P *= 0.0005) (Figure 4). In the subgroup of patients not colonised at admission, a statistically significant increase in the CCI was also observed in group C as compared with group N at T9 (median 0.14 vs. 0.00, respectively; *P *= 0.04), but this significance did not persist over time and the very low number of the patients did not allow any analysis.

No fungal infection was recorded, even in heavily colonised patients. No clinically adverse effect related to nystatin therapy was recorded.

## Discussion

The main finding of the present study is that systematic nystatin prophylaxis significantly reduces the development of *Candida *species colonisation and that it significantly reduces the CCI after 6 days of treatment in patients already colonised at ICU admission.

Invasive candidiasis infections are associated with an increased morbidity and mortality in ICU patients (30 to 40%), depending on the severity of underlying disease, the *Candida *species involved, and the timing and choice of antifungal treatment [1]. Early and adequate antifungal treatment is well known to be independently associated with a reduction of hospital mortality [20]. This is the reason why early antifungal treatments, such as prophylaxis, pre-emptive, or empiric therapies, have progressively emerged; however, most of these strategies lack an evidence base that would establish them as the standard of care. Several studies evaluated the effects of prophylaxis or pre-emptive therapy on fungal infection and mortality, and most of them compared fluconazole with placebo [21,22]. Some concerns remain about the use of azoles for prophylaxis, including the emergence of resistance among previously susceptible strains (*C. albicans*) or a shift to less susceptible or resistant non-*albicans *species (*C. glabrata *or *C. krusei*), increased costs, and adverse events [23,24]. Moreover, because patient populations, dosing regimens, severity of illness, and definitions of infection and other outcome measures varied between trials, conclusions are difficult to extrapolate. Current recommendations limit the use of antifungal prophylaxis to high-risk adult patients admitted to those ICUs that have very high rates of invasive candidiasis, and advocate against the widespread use of an antifungal in all ICU patients [25,26].

In this context, the use of nystatin may present a rationale alternative [27]. Nystatin, like amphotericin B, is a nonabsorbable polyene with a wide antifungal activity, especially against *Candida *species, including *C. glabrata *and *C. krusei*. Although most species of *Candida *isolated in ICU patients remain susceptible to azoles, indiscriminate use of such drugs may lead to the spread of *C. krusei *and *C. glabrata*, intrinsically resistant to and dose-dependently sensitive to azoles, respectively [28]. In contrast, primary resistance to polyenes among *Candida *species is limited to *Candida lusitaniae *and to some strains of *Candida guillermondii*, and resistance seldom develops during treatment [29]. Moreover, no adverse effects of nystatin are reported. Azoles are generally well tolerated, but side effects such as hepatic dysfunction are possible. These risks, even if low, could be more difficult to accept in the setting of prophylaxis in critically ill patients. The third advantage of oral nystatin is its low cost, making this strategy potentially highly cost-effective.

Nonabsorbable polyenes are integrated in most selective decontamination of the digestive tract regimens, and a recent meta-analysis showed that they significantly reduce fungal carriage and overall fungal infections, but without impact on fungemia [30]. This meta-analysis, however, included only two trials testing nystatin prophylaxis. Moreover, the majority of selective decontamination of the digestive tract regimens included either concurrent systemic or topical antibacterial antibiotic prophylaxis in the treatment group, which might increase the risk of fungal infection in that group relative to the placebo group. A recent study investigated the effect of oral nystatin prophylaxis to prevent *Candida *species colonisation [16]. This trial, however, included both medical and surgical patients and excluded those patients who exhibited baseline *Candida *species colonisation. Since colonisation can be observed on admission to the ICU in almost 50% of patients [17,18], a considerable cohort of ICU population was excluded in this latter study, making these positive results not applicable to all ICU patients.

The present trial showed that oral nystatin prophylaxis started on the day of admission is significantly effective in reducing heavy fungal colonisation, even in baseline colonised patients. This finding was presumably related to the efficacy of oral nystatin to significantly reduce the proportion of positive gastrointestinal sites (for example, stomach and rectum), as shown in the treatment group. Accordingly, the CCI regularly decreased over time in patients receiving nystatin, whereas it tended to increase in controls. No adverse events developed during the study period, confirming that this polyene is a safe choice in the surgical ICU population. Interestingly, nystatin prophylaxis also reduces colonisation in the urinary tract, which is now advocated as the easier and simpler marker for heavily colonised patients [31]. A possible explanation could be that oral nystatin, by reducing gastrointestinal fungal carriage, can also help to control genital and perineal fungal colonisation, thus lowering the risk of retrograde access to the urinary tract, especially in the presence of indwelling bladder catheters. Moreover, oral nystatin increases costs only by €1.1/patient/day of treatment (data not shown).

In the present study, almost 70% of included patients were colonised at admission. This was a surprising finding. While neurosurgical and abdominal surgery patients have several comorbidities and risk factors (that is, long hospital stay, central venous catheter, parenteral nutrition, antibiotic therapy) that may predispose to subsequent *Candida *colonisation, this is usually not true for trauma patients. When we analysed the subgroup of trauma patients, however, we noted that almost 50% of them were colonised at admission to the ICU - suggesting that, in many cases, fungal colonisation can be community acquired and not only hospital related. Further trials are needed, in our opinion, to investigate this hypothesis.

*Candida *colonisation is very common among ICU patients, reaching 60% in non-neutropenic critically ill patients [32], and is a well-known risk factor for invasive candidiasis [9,13] since changes in the ecology of the endogenous flora may promote *Candida *species overgrowth on mucosal and skin surfaces [11] and translocation across the gut barrier, mostly when its integrity is lost [12,33]. *Candida *colonisation can be statistically associated with a higher frequency of clinical manifestation or even higher mortality [14]. Based on the previous consideration, we suggest the use of nystatin for fungal pre-emptive therapy in high-risk colonised patients on admission to the ICU as a rationale choice, since it could be effectively used in almost all ICU patients with CCI > 0.4 without increased risk of adverse events. Calculating the CCI, however, is time consuming and resource consuming and is not always feasible. When the CCI is not known we favour nystatin use in those patients expected to require a long ICU stay. Only under these conditions will the risk-benefit and cost-benefit ratios for prophylaxis reflect an advantage for the patient [34].

Unfortunately, no definitive conclusion regarding the effect of nystatin prophylaxis on *Candida *infection can be drawn from our study because none of the included patients, even if heavily colonised, developed the infection. A probable hypothesis to explain this unexpected result is that, in our ICU, a rigid surveillance policy of central venous catheters was undertaken, including strict asepsis during insertion, careful medication, and early removal as soon as possible (median 3 days). This approach, together with a rapid interruption of parenteral nutrition in favour of the enteral route, could justify why no episode of *Candida *infection was documented during the study period. Moreover, the number of patients with abdominal surgery is low, especially in comparison with neurosurgical patients. This factor could be important because abdominal surgery is a risk factor for invasive candidiasis more than other types of surgery. This trial, finally, was designed to investigate the effect of nystatin prophylaxis on fungal colonisation during the ICU stay and not to detect any reduction in fungal infection, which would have required a larger number of patients.

Other limitations of the present study included the single-centre, open-label design and the small sample size. The inclusion criteria adopted (surgical patients expected to require invasive mechanical ventilation for more than 48 hours), however, selected a homogeneous population of ICU patients, treated by the same staff adopting the same protocols to prevent infection - assuring, in our opinion, consistent and reliable results. Larger trials are warranted to elucidate the impact of nystatin prophylaxis on the infection rate and mortality in critically ill patients.

## Conclusion

The present trial shows that nystatin pre-emptive therapy in surgical/trauma ICU patients significantly reduces fungal colonisation, even in those colonised at admission. Moreover, when CCI is not calculated, we suggest nystatin use in those patients expected to require a long intensive care stay. This approach could contain antifungal therapy costs. Further clinical data are needed, however, to better identify patients who might warrant antifungal prophylaxis using drugs with low risk of resistant stain emergence, in order to drastically reduce fungal-related morbidity and mortality.

## Key messages

• The incidence of nosocomial candidemia has dramatically increased and has been associated with high overall (35 to 80%) and attributable (30 to 40%) mortality.

• An early and adequate antifungal treatment is independently associated with a reduction of hospital mortality.

• Oral nystatin prophylaxis started at the day of admission is significantly effective in reducing fungal colonisation, even in baseline colonised ICU patients.

## Abbreviations

CCI: corrected colonisation index; CI: colonisation index; group C: control group; group N: nystatin group; T0: day of admission to the ICU; T3: third day of ICU stay; T6: sixth day of ICU stay; T9: ninth day of ICU stay; T12: 12th day of ICU stay; T15: 15th day of ICU stay.

## Competing interests

The authors declare that they have no competing interests.

## Authors' contributions

MG participated in the design of the study, acquisition of data, drafted the manuscript, and performed statistical analysis. GC participated in the design of the study and acquisition of data. LD participated in the design of the study and performed statistical analysis. NB drafted and revised the manuscript for important intellectual contents. IA participated in the design of the study, acquisition of data, and drafted the manuscript. AS, AF, and CC participated in the acquisition of data. MTM contributed to analysis and interpretation of data. FB conceived the study and approved the final version to be published. FP drafted the manuscript, contributed to analysis and interpretation of data, and revised the manuscript. All authors read and approved the final manuscript.
